# Acute Hæmatemesis

**Published:** 1858-04

**Authors:** B. F. Schenck

**Affiliations:** Lebanon, Pa.


### ARTICLE II.

### ACUTE HÆMATEMESIS; DEATH ON THE EIGHTEENTH DAY; ULCERS ON THE MUCOUS MEMBRANE OF THE STOMACH.

BY B. F. SCHENCK, M.D., LEBANON, PA.

1855. Kathinka Weber, aged sixteen years, is the daughter of German parents, living in this country about three years. The father is perfectly healthy; but the mother has a complete tubercular diathesis, and is a regular, standing patient. Three or four times a year she gets scrofulous inflammation of the lungs; has an incurable albuminuria; and, as though this were not enough, she has, every three or four weeks, either a violent menorrhagia, an agonizing facial neuralgia, or an attack of spasm—of an hysterical nature—affecting every muscle of the body, during which she is as rigid as a board, and wholly unconscious, often for a period of six or twelve hours. From all these various attacks—delicate woman as she appears and actually is—it is always a matter of surprise that she ever recovers. There are two boys, aged seven and twelve years, rather pale and sickly-looking, the younger of whom, for four months after leaving the ship, had incessant vomiting—a sort of chronic sea-sickness; and two girls, the eldest about twenty years of age, both of them very fresh and rosy.

During the cherrying-time, Kathinka, being out with some young companions, fell from a tree, a height of twelve or fifteen feet, but was so little hurt that she walked home, a distance of three miles. After this, her only complaint was of pain across the stomach, not severe, but persistent. She, however, kept a good color, and had an excellent appetite, as usual.

On the afternoon of August 22d, she suddenly felt sick at stomach, and before she could help herself, threw up nearly two pints of liquid blood and coagula, of a dark color. She vomited twice more on the same afternoon, and each time fully a pint. After the prostration incident to the hemorrhage had passed off, she became highly feverish, complained of pain in the hepatic region, and had a furred tongue, with great thirst. Without thinking immediately of the fall, I attributed the symptoms to hepatic or splenic congestion; and the full, vigorous pulse appearing to indicate V. S., I drew Oi. blood, gave 10 grs. calomel, to be followed by sulph. magnesia, and allowed ice freely. She now began to improve, when, on the morning of the 26th, she again vomited three times in the course of an hour, and very copiously; constant nausea; the following night passed several dark, tarry stools; on 28th, vomited again twice, very largely, coagula looking quite black. Since the 26th, has been taking tinct. chlorid. iron; on 28th, substituted pb. acetat. 1 gr., opium, $\frac{1}{8}$ gr., every hour or two.

29, 30, 31. No vomiting, but constant nausea. Epispastic to stomach, and morphia endermically. Evening of 31st, vomited once; saw her in consultation with Dr. Marshall. The liquid just ejected did not look at all like blood, but like a disorganized animal fluid—dark and dissolved. Floating in this liquid, which amounted to nearly a pint, was a large, white, ovoid mass, about two in. long by $\frac{3}{4}$ in. thick, smooth and pretty firm, along with a number of smaller whitish particles of the same kind. At first sight, it resembled coagulated milk; but the patient had not taken any milk for several days. It was also much more dense, approaching the cerebral substance in character. After close scrutiny, we pronounced it to be either encephaloid matter, or a softening tubercular mass. But whence could it come? The only probable source would be the liver. How? Perhaps through an abscess of this organ communicating with the stomach; but of this we have no evidence, the patient having discharged no pus either way. Perhaps the fall has lacerated the liver, which, being glued by the adhesive inflammation to the stomach, would offer this intercommunication. But admitting all this, and assuming this mass to be tubercular matter, would so large a deposit of morbid products as this sample necessarily presupposes, be consistent with the state of perfect health which the patient enjoyed up to the moment of attack?

*Sept.* 1. Vomited again once, this morning, a dark liquid, resembling that of the previous evening. Acid. sulphuric. aromat., twenty drops every two hours.

*Sept.* 2. No vomiting; constant nausea and retching. Bowels not moved for three days past; turpentine enema.

*Sept.* 3. Vomited this morning about half a pint of coagula, of a bright red color, showing the blood to have been of recent effusion, and not yet acted on and colored by the gastric juice. Completely foiled, I returned again to acet. lead and opium. Nausea all day; turpentine enema, but no motion.

*Sept.* 4, 5. Incessant retching and nausea; brings up occasionally a little bile. Has a craving for hot drinks. Gave creosote, one drop every three hours. Obstinate constipation, in spite of repeated enemata. Very severe pain in the stomach, which is somewhat tumid.

*Sept.* 6, 7, 8. Gradually getting worse. Abdomen tympanitic. On morning of 7th, had five greenish, inodorous, copious passages. Abdominal pain and distension very much increased;

"felt as though she must burst." Died on the morning of the 8th, with her mind clear to the last moment.

*Autopsy, twelve hours after death.*—The abdomen was extremely tumid and tense. Proceeding to raise a triangular flap, from the sternum down towards the crest of the ilium on each side, upon the first incision into the peritoneal cavity on the left side, a large gush of yellowish serum escaped. Upon completing the division, a voluminous mass of intestine burst through the opening, consisting of colon, jejunum and ileum. Looking naturally first for the stomach, it was found closely pressed up against the diaphragm. Removing it from the body, it was seen to contain several ounces of green liquid, apparently more than half bile. The coats of the organ were very much thickened—feeling not unlike the softest buckskin leather, and of a dull white color. The folds of the mucous membrane were very prominent; and the posterior aspect of the stomach presented, over a considerable portion of its mucous surface, a very minutely speckled appearance, resembling, as observed by Andral, that produced by scattering fine grains of powdered vermilion upon a moist sheet of white paper. These dots were very closely set together, and may have been one source of the hemorrhage, if we suppose the blood to have escaped by exudation, in consequence of great capillary congestion. Directly opposite, upon the mucous membrane of the anterior surface, were several ulcers, in various stages of progress. Two, especially, looked quite recent, appearing as though the mucous membrane had been dug out, and were of the diameter of a goose-quill. Another of the same size, but older, was directly in the course of a small bloodvessel, and had a bluish color, and a somewhat glazed appearance, as though cicatrization had commenced.

The liver was next examined. Beyond a considerable enlargement and congestion of the organ, there was no evidence of disease or injury. The gall-bladder was very full of bile; and the transverse colon, where it rests below the liver, looked as if stained, of a bright chocolate, or tinct. iodine color. The spleen presented no appreciable lesion. The heart was healthy. The lungs showed no trace of tubercles, and upon incision exuded merely a reddish, frothy serum.

Since the death, I have been told by the parents, that when the deceased girl and her elder sister were about the ages of five and nine years, the following incident occurred. Friction matches were kept for sale in the house; and the warmth of the sun through the window glass, it was supposed at the time, had caused the phosphorus compound to soften, and run from the matches on to the paper in which they were wrapped. The composition from at least as many as two of our ordinary match boxes full, I have been assured, had thus accumulated upon the wrappers, and being found by these children, was eaten by them for sugar. They, however, soon sought their mother, to tell her that "it was queer sugar, for it burned them." No vomiting was produced; sweet milk was freely given, and as the children did not sicken, no further attention was paid to the matter. But, I am told that both girls have since then had much complaint to make of pain and epigastric uneasiness; and the surviving girl was under my care last winter for an attack of severe epigastric pain with great swelling, which yielded mainly to poultices and tart. em. oint. Can the phosphorus eating be regarded as the cause of these ailments, and of the death?

